# High Systolic and Diastolic Blood Pressure Variability Is Correlated with the Occurrence of Peripheral Arterial Disease in the First Decade following a Diagnosis of Type 2 Diabetes Mellitus: A New Biomarker from Old Measurement

**DOI:** 10.1155/2016/9872945

**Published:** 2016-10-18

**Authors:** Chi-Hsiao Yeh, Hsiu-Chin Yu, Tzu-Yen Huang, Pin-Fu Huang, Yao-Chang Wang, Tzu-Ping Chen, Shun-Ying Yin

**Affiliations:** ^1^Department of Thoracic and Cardiovascular Surgery, Chang Gung Memorial Hospital, Keelung, Taiwan; ^2^College of Medicine, Chang Gung University, Tao-Yuan, Taiwan; ^3^Department of Nursing, Chang Gung Memorial Hospital, Keelung, Taiwan

## Abstract

*Background*. To assess whether the visit-to-visit variability in blood pressure (BP) is a risk factor of peripheral arterial disease (PAD) in patients with type 2 diabetes mellitus (T2DM) 10 years after diagnosis.* Methods*. The electronic medical records of 825 patients, who were diagnosed with type 2 diabetes mellitus (T2DM) during 2000–2002 and regularly followed for 10 years, were retrospectively reviewed. A total of 53,284 clinic visit records, including analysis of BP, BMI, serum glycohemoglobin, and lipid profile, were analyzed.* Results*. Patients were categorized into two groups according to their visit-to-visit variability in systolic and diastolic BP (SBP and DBP, resp.). The high-risk group included patients with high SBP and DBP visit-to-visit variability; this group had a 1.679-fold (95% CI: 1.141–2.472, *P* = 0.009) increased risk of PAD compared with patients in the low-risk group. Cox regression analysis also demonstrated that the age at which the patients were diagnosed with T2DM, smoking status, and mean creatinine level was significantly associated with increased risk of PAD with a hazard ration of 1.064 (95% CI: 1.043–1.084, *P* < 0.001), 1.803 (95% CI: 1.160–2.804, *P* = 0.009), and 1.208 (95% CI: 1.042–1.401, *P* = 0.012), respectively.* Conclusions*. High SBP and DBP visit-to-visit variability is correlated with PAD in the first decade following a diagnosis of T2DM.

## 1. Introduction

In patients with diabetes mellitus, peripheral arterial disease (PAD) is a major risk factor for lower-extremity amputation [1]. However, it is difficult to determine the prevalence of PAD in patients with diabetes given its asymptomatic characteristic, the diverse screening modalities employed, and blunted pain sensation due to peripheral neuropathy, resulting in its underestimation [1]. The ankle-brachial index (ABI), the ratio of the systolic blood pressure (SBP) measured at the ankle to that measured at the brachial artery [2], has a sensitivity of 95% and specificity of almost 100% for PAD diagnosis, when validated against angiographically confirmed disease [3]. In diabetic patients older than 40 years of age examined using the ABI, the prevalence of PAD was 20% [4]. In contrast, Hirsch et al. [5] reported a prevalence of PAD of 29% in diabetic patients older than 50 years of age.

Identifying the biomarkers for PAD is important for development of prevention modalities in diabetic patients, especially given that the 5-year cardiovascular event rate, including nonfatal myocardial infarction (MI) and stroke, for PAD patients with T2DM was 20% and the mortality rate was 30% [6]. Moreover, after adjusting for risk factors, patients with PAD had a twofold increased risk of MI, stroke, and mortality rate [7]. Moreover, 27% of patients with PAD demonstrate progression of symptoms with 4% experiencing limb loss after 5 years [8].

A diagnosis of PAD can identify patients who have higher risk of subsequent MI or stroke, and treating hypertension in PAD patients reduces the risk of MI, stroke, heart failure, and death [9]. Diagnosis and treatment of symptomatic PAD patients with a supervised exercise program and cilostazol may improve the quality of life and prevent functional disability and limb loss as well [10]. Both the American Diabetes Association guidelines [11] and the seventh report of the Joint National Committee on Prevention, Detection, Evaluation, and Treatment of High Blood Pressure [12] recommend using absolute BP as a therapeutic target to prevent clinical stroke and heart disease, as well as PAD with paucity of evidence [13]. However, no randomized prospective clinical trial has conclusively proven the benefits of treatment in individuals with stage 1 systolic hypertension [12].

Although mean BP values are largely considered the cause of the adverse cardiovascular consequences associated with hypertension, the possible role of increased BP variability has also been reported in observational studies and clinical trials [14–17]. Specifically, the visit-to-visit variability of systolic BP (SBP) had been shown to be a novel biomarker for the development of stroke and coronary artery diseases [14–19], the progression of a carotid artery stenosis and peripheral vascular disease [20–22], and the deterioration in renal function for stages 3-4 diabetic chronic kidney disease (CKD) patients [23]. However, little is known about the long-term association of BP visit-to-visit variability with PAD occurrence in diabetic patients with normal ABI at diagnosis.

The association between BP visit-to-visit variability and cardiovascular events generally considers BP measurements at a few time points and in a short to medium follow-up period, limiting the appreciation of the full impact of BP variability on PAD, especially for diabetic patients. Therefore, we evaluated the long-term relationship between BP visit-to-visit variability and the occurrence of PAD in patients from the beginning of their diagnosis with type 2 DM (T2DM).

## 2. Materials and Methods

### 2.1. Patients and Study Design

We retrospectively collected the following 10-year measurements obtained at every outpatient clinic visit of 825 patients who were first diagnosed with T2DM during 2000–2002 at Chang Gung Memorial Hospital, Keelung: blood pressure, body weight, body height, and laboratory data. T2DM was diagnosed in accordance with the criteria of the American Diabetes Association [24]. Body mass index (BMI) was defined as weight (kilograms) divided by height (meters) squared. Patients were classified as nonsmokers, former smokers, or current smokers according to the electronic medical record. PAD was diagnosed on the basis of an ABI ≤ 0.9 [25]. Patients who developed PAD or cardiovascular disease (CVD), including coronary artery disease, MI, ischemic stroke, or transient ischemic attack [26], before being diagnosed with diabetes were excluded. Patients who never had an ABI assessment during the 10-year follow-up period, those without PAD who did not have complete ABI data in the 10th year of follow-up, and patients with less than 10-year follow-up were also excluded. Dyslipidemia was defined as without treatment, total cholesterol levels >200 mg/dL, low-density lipoprotein cholesterol levels >100 mg/dL, high-density lipoprotein cholesterol levels <50 mg/dL in females and <40 mg/dL in males, or triglycerides >150 mg/dL. Hypertension was defined as SBP ≥130 mmHg or diastolic blood pressure (DBP) ≥80 mmHg in diabetic patient before any treatment was initiated [12].

This study was conducted in accordance with the Declaration of Helsinki and was approved by the Institutional Review Board of Chang Gung Memorial Hospital; informed consent was waived.

### 2.2. Laboratory Assessments

BP measurements at every visit were recorded throughout the follow-up period till PAD was diagnosed. All outpatient clinics used automated sphygmomanometers operated by trained medical assistants after 10–15 minutes resting, with repeated measurements performed as needed by physicians using aneroid sphygmomanometers [27]. Fasting serum total cholesterol, low-density lipoprotein, high-density lipoprotein, and triglyceride concentrations were assessed using standard enzymatic methods. Hemoglobin A_1c_ was assayed using high-performance liquid chromatography and expressed with the unit defined by the National Glycohemoglobin Standardization Program.

### 2.3. Definition of BP Visit-To-Visit Variability

BP visit-to-visit variability and the coefficient of variation (standard deviation (SD) of mean BP divided by mean BP) of SBP and DBP were determined [28]. The BP instability index was expressed as the delta BP, which was defined as a difference between the maximum BP and the minimum BP, through all visits till PAD was diagnosed [29].

### 2.4. Statistical Analysis

Means and frequencies of potential confounding variables were calculated. The relationships between variability in SBP and DBP, as well as other variables, and PAD were examined by Pearson's correlation analyses. To examine the effects of various factors on the occurrence of PAD, the following factors were considered simultaneously as independent variables for Cox multiple regression analysis: age at DM diagnosis, sex, BMI, average SBP and DBP, SD of SBP and DBP, maximum of SBP and DBP, delta SBP, delta DBP, hemoglobin A_1c_, total cholesterol, triglyceride, smoking status, presence of CVD, hypertension, and dyslipidemia. All continuous variables are presented as the mean ± SD or absolute number. A *P* value < 0.05 was considered statistically significant. The area under each receiver operating curve and 95% confidence intervals (CI) were estimated to compare the relative ability of the SD of SBP and DBP to identify risk of peripheral arterial disease in diabetic patients. The optimal cut-point BP was calculated based on the Youden Index [30], which was calculated as sensitivity + specificity − 1 [31]. Multicollinearity was assessed using variance inflation factor (VIF) among average, SD, maximum, and grouping of VVV of SBP and DBP [32, 33]. The power calculation was performed with pass software. Multicollinearity was diagnosed with a VIF, one of the most common tools used by statisticians, of 5 and above [32, 33].

## 3. Results

Nine hundred and thirty-six patients were first diagnosed with DM at Chang Gung Memorial Hospital from 2000 to 2002. Sixty-nine patients who died or were lost to follow-up were excluded. Twenty-three patients without final ABI data and 19 patients who had PAD or CVD before their DM diagnosis were also excluded. The characteristics of the 825 patients enrolled in this study are shown in Table 1. The overall mean age at diagnosis with DM was 53.6 ± 10.5 years. At baseline, the ABI for all patients was in the range of 0.9–1.3. The median observation period was 148.1 ± 16.0 months. At the end of the observation period, the right and left leg ABI levels for all patients were 1.02 ± 0.24 and 1.02 ± 0.42, respectively. There were 114 patients diagnosed with PAD during the 10-year follow-up with an average time of PAD diagnosis of 116.7 ± 12.8 months after their DM diagnosis. The average SBP and DBP for all patients was 136.8 ± 10.2 and 73.5 ± 6.4 mmHg, respectively. The SD of the SBP and DBP for all patients was 14.8 ± 3.8 and 7.5 ± 2.1 mmHg, respectively.

Multivariate Cox regression analyses revealed that the SD of SBP was positively correlated with the occurrence of PAD (*P* = 0.037; Table 2). However, the maximum of SBP and delta of SBP were not significantly correlated. The mean, SD, maximum, and delta of DBP were not significantly correlated with the occurrence of PAD. In addition to the SD of SBP, the age at DM diagnosis was positively correlated with the occurrence of renal function impairment (*P* < 0.001, HR = 1.064, 95% CI = 1.043–1.084). In addition, the occurrence of PAD was associated with mean creatinine level (*P* = 0.012) and current smoking status (*P* = 0.009). However, mean or SD of hemoglobin A_1c_, BMI, total cholesterol, low-density lipoprotein, high-density lipoprotein, and triglyceride were not independently correlated with the occurrence of PAD (Table 2).

We next categorized the patients into high- or low-risk groups on the basis of their SD of SBP or DBP. Cut-off points for the SD of SBP and DBP, where the sensitivity approximates specificity for the occurrence of PAD, are 16.3 and 7.6 mmHg, respectively. Patients with SD of SBP and DBP higher than the cut-off values (*n* = 199) were placed in the high-risk group, and all the other patients (*n* = 626) were in the low-risk group. The multicollinearity was assessed between VVV grouping and mean, SD, maximum, and delta of SBP and DBP. The VIFs of all these factors were less than 2 (Table 2), which represented the idea that the grouping according to the VVV was independently different factor among these parameters. The characteristics of both groups were shown in Table 3. The age at DM diagnosis, hypertension history, SD of BMI, average SBP and DBP, SD of SBP and DBP, delta SBP and DBP, mean and SD of hemoglobin A_1c_, mean total cholesterol, and mean and SD of creatinine level were significantly different between the low- and high-risk groups (*P* ≤ 0.034).

**Figure 1:**
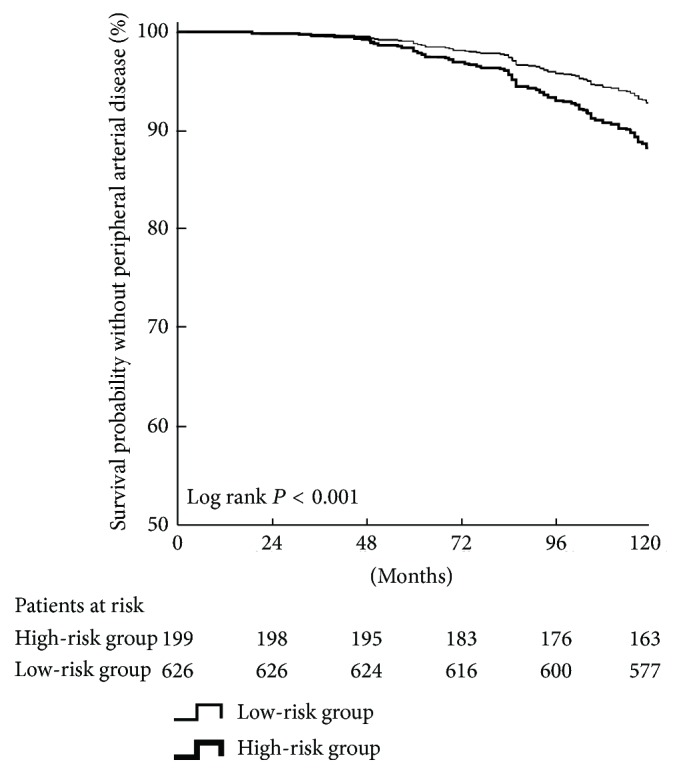
Kaplan-Meier plot of peripheral arterial disease occurrence over 10 years following a diagnosis of type 2 diabetes. Patients were grouped into high- (high BP visit-to-visit variability) and low-risk groups.

In the 10 years following their DM diagnosis, 50 patients (25.1%) in the high-risk group had PAD versus 64 patients (10.2%) in the low-risk group (*P* < 0.001; Table 3). The PAD-free survival curve between patients in high- and low-risk groups was shown in Figure 1. In addition, the occurrence of CVD in the high-risk group was significantly higher than that of the low-risk group (25.1% versus 11.7%; *P* < 0.001).

Cox multivariate regression analysis revealed that the risk of PAD increased by 1.064-fold as the age at diagnosis increased by 1 year (*P* < 0.001, 95% CI 1.043–1.084; Table 4). High BP visit-to-visit variability also increased likelihood of PAD within 10 years of being diagnosed with DM by 1.679-fold (*P* = 0.009). Current smoking status and elevation of mean creatinine level was also associated with increased risk of PAD (Table 4).

## 4. Discussion

This study showed that patients with high visit-to-visit variability in both SBP and DBP were more frequently diagnosed with PAD in the first decade following diagnosis with DM. However, the mean and delta SBP/DBP, mean serum lipid profile, mean and SD of hemoglobin A_1c_ concentration, and BMI were not correlated with the occurrence of PAD. Our results also confirmed that old age at DM diagnosis, smoking, and renal function impairment would increase the risk of PAD in the first decade after DM was diagnosed. Cessation of smoking in patients with PAD could substantially reduce the risk of death, myocardial infarction, and amputation and increase the patency rate of lower-extremity angioplasty and surgical revascularization [25]. Two recent studies revealed that central obesity with elevated BMI was positively associated with ABI increase [34, 35]. However, the relationship between BMI and PAD could not be identified from our study.

Assessment of the effects of short-term variability of BP has traditionally dominated this field of research [36] and diminished the interest in long-term variability of BP, such as those occurring between days or months. However, recent studies have shown that long-term visit-to-visit variability of BP may have greater prognostic value than mean BP or short-term variability [19, 37, 38]. These studies recommended that optimal antihypertension treatment included avoidance of inconsistent BP control and large BP visit-to-visit variability [36].

The effect of high BP variability on PAD occurrence has rarely been studied. Most prospective BP variability studies focused on the cardiovascular mortality and morbidities, including MI, stroke, and heart failure [17, 39–41]. These studies followed patients for a relatively short period of 1.5 to 7.8 y, and less than 10% of the study patients had DM [39–41]. DM is one of the strongest risk factors for critical limb ischemia and amputation, as well as incident PAD in population studies [42]. However, few studies have completely focused on the detrimental cardiovascular effect of BP variability in diabetic patients. Mancia et al. [43] revealed that DM did predict ABI decline over an average of 4.6 y of follow-up. Our study proved that high variability of BP is a risk factor for the occurrence of PAD in the 10 years following DM diagnosis. These diabetic patients with PAD are at high risk for adverse cardiovascular events unless the PAD is recognized. From the results of the present study, diabetic patients with high BP visit-to-visit variability had a 1.679-fold increased risk of the occurrence of PAD. For the first time, our results demonstrated that a visit-to-visit variability VVV of SBP > 16.3 mmHg with a visit-to-visit variability of DBP > 7.6 mmHg significantly increased the risk of PAD in the first decade following a diagnosis with DM. The adverse consequences of high BP variability on the cardiovascular system might result from the traumatic effect of large blood pressure oscillations enhancing the intravascular pressures on the vessel wall, promoting tissue growth and atherosclerosis [44].

Office BP as the measurement of variability of BP may be limited due to white coat hypertension although this issue is controversial. A meta-analysis that included 7961 untreated participants reported that the cardiovascular risk was not significantly different between white coat hypertension and normotension [45]. Another study from the International Database of HOme blood pressure in relation to Cardiovascular Outcome, which followed 6458 patients for a median of 8.3 y, reported that the cardiovascular risk for the white coat hypertension did not differ from that of the treated participants [46]. However, another large meta-analysis of 29,100 patients comparing white coat hypertension participants with sustained hypertension participants found that CVD mortality (4.2%) was significantly lower in white coat hypertension participants (6.6%; OR = 0.47, 95% CI 0.35–0.64; *P* < 0.001) than those with sustained hypertension participants [46]. Thus, when office BP is used as the measurement of BP variability, white coat hypertension is not a factor that induces bias.

The retrospective nature of the present study and its sample size are two of the limitations of the present study. The possibility of type 2 error exists. Another limitation is the medication record through the 10-year follow-up period. Antihypertensive drug use in each patient was not consistent throughout the 10-year follow-up period. Thus, it is very difficult to clarify the effect of BP visit-to-visit variability amelioration by each category of antihypertensive drug, such as calcium channel blockers, which had a controversial effect on blunting the amplitude of BP visit-to-visit variability [23]. The other limitation of the present study is the absence of antihypertensive prescription fill data and information regarding patients' adherence to treatment regimens. However, low antihypertensive medication adherence explained only a small proportion of BP visit-to-visit variability [38], which implied that the absence of medication adherence data does not have a major impact on the results of the present study.

Despite the aforementioned limitations, this study has several strengths, including the long follow-up period to assess the occurrence of PAD and the available information on demographic, clinical, and long-term BP data. In addition, the use of an electronic medical record database provided real-world evidence on the status of hypertension control in the first decade following a diabetes diagnosis and minimizes selection bias related to self-selection into the study. Prospective studies are in need in verifying the high variability of SBP visit-to-visit as early biomarker for detection of PAD in the first decade following the diagnosis of T2DM. More research is needed to fully understand the association between BP visit-to-visit variability and risk of vascular events in diabetic patients, and large-scale pooled analyses of multiple cohorts will be required [19].

## 5. Conclusion

The present study showed that, in diabetic patients with initially normal ABI values, high BP visit-to-visit variability was a significant early biomarker for detection of PAD in the first decade following the diagnosis of T2DM.

## Figures and Tables

**Table 1:** Characteristics of the study participants (*n* = 825).

Characteristic	
Age at DM diagnosis (y)	53.6 ± 10.5
Sex (male, %)	390 (47.3)
Smoking status (none/former/current)	628/49/148
Hypertension (%)	629 (76.2)
Hyperlipidemia (%)	793 (96.1)
Body mass index (kg/m^2^)	26.8 ± 3.9
Number of measurements	45.0 ± 24.8
Average SBP (mmHg)	136.8 ± 10.2
Number of measurements	63.4 ± 29.4
SD of SBP (mmHg)	14.8 ± 3.8
Maximum of SBP (mmHg)	174.5 ± 17.5
Delta SBP (mmHg)	72.5 ± 25.6
Average DBP (mmHg)	73.5 ± 6.4
SD of DBP (mmHg)	7.5 ± 2.1
Maximum of DBP (mmHg)	93.8 ± 11.3
Delta DBP (mmHg)	38.2 ± 14.6
Hemoglobin A_1c_ (%)	7.6 ± 1.0
Number of measurements	34.6 ± 10.9
Total cholesterol (mg/dL)	193.5 ± 28.6
Number of measurements	11.9 ± 6.1
High-density lipoprotein (mg/dL)	38.5 ± 10.6
Low-density lipoprotein (mg/dL)	118.5 ± 20.0
Triglyceride (mg/dL)	150.1 ± 112.1
Initial creatinine (mg/dL)	0.76 ± 0.54
Average creatinine (mg/dL)	0.99 ± 0.71
Number of measurements	17.9 ± 6.7

Clinical events during the 10-year follow-up	
PAD (%)	114 (13.8)
CVD^a^ (%)	123 (14.9)
CAD or MI (%)	38 (4.6)
TIA or stroke (%)	88 (10.7)
Total follow-up period (months)	148.1 ± 16.0

DM, diabetes mellitus; SBP, systolic blood pressure; DBP, diastolic blood pressure; SD, standard deviation; PAD, peripheral arterial disease; CVD, cerebrovascular disease; CAD, coronary artery disease; MI, myocardial infarction; TIA, transient ischemic attack; CKD, chronic kidney disease.

^a^Defined as cerebrovascular disease, neurodegenerative disease, and Parkinson's disease that required medical treatment and long-term follow-up.

**Table 2:** Multivariate Cox regression analyses of the factors associated with peripheral arterial disease (*n* = 825) in the 10 years following a diagnosis of DM.

Independent variable	Hazard ratio	95% CI	VIF	*P* value
Sex (female = 0)	0.893	0.558–1.429		0.636
Age at DM diagnosis	1.069	1.043–1.095		<**0.001**
Nonsmoking				**0.026**
Former smoker	1.627	0.765–3.460		0.206
Current smoker	2.070	1.212–3.536		**0.008**
Hypertension	1.426	0.733–2.774		0.296
Dyslipidemia	4.124	0.543–31.319		0.171
Mean SBP	1.008	0.974–1.044	1.420	0.639
SD of SBP	1.088	0.970–1.220	1.816	**0.150**
Maximum of SBP	0.984	0.960–1.008	1.267	0.182
Delta of SBP	1.001	0.986–1.015	1.806	0.932
Mean DBP	0.996	0.936–1.060	1.460	0.899
SD of DBP	0.940	0.757–1.168	1.859	0.578
Maximum of DBP	1.015	0.982–1.049	1.267	0.364
Delta of DBP	0.997	0.971–1.023	1.779	0.809
Mean BMI	0.972	0.917–1.030		0.338
SD of BMI	1.251	0.868–1.802		0.230
Mean hemoglobin A_1c_	0.979	0.764–1.254		0.864
SD of hemoglobin A_1c_	0.979	0.565–1.695		0.940
Mean serum cholesterol	1.011	0.998–1.023		0.088
SD of serum cholesterol	0.994	0.983–1.005		0.258
Mean serum LDL	1.004	0.990–1.018		0.565
SD of serum LDL	0.988	0.976–1.001		0.079
Mean serum HDL	0.993	0.968–1.018		0.575
SD of serum HDL	1.022	0.980–1.067		0.309
Mean serum triglyceride	1.001	0.996–1.005		0.815
SD of serum triglyceride	1000	0.995–1.004		0.892
Mean creatinine	1.305	1.024–1.665		**0.032**
SD of creatinine	0.833	0.474–1.461		0.523

DM, diabetes mellitus; SBP, systolic blood pressure; DBP, diastolic blood pressure; SD, standard deviation; CV, coefficient of variation; BMI, body mass index; LDL, low density lipoprotein; HDL, high density lipoprotein; VIF, variance inflation factor.

**Table 3:** Demographics and clinical characteristics of the low- and high-risk groups as determined by BP visit-to-visit variability.

	Low-risk group (*n* = 626)	High-risk group (*n* = 199)	*P* value
Age at DM diagnosis (y)	52.5 ± 10.3	57.2 ± 10.6	<**0.001**
Sex (male, %)	296 (47.3)	94 (47.2)	0.528
Smoking (none/former/current)	480/36/110	148/13/38	0.808
Hypertension (%)	449 (71.7)	180 (90.5)	<**0.001**
Hyperlipidemia (%)	600 (95.8)	193 (97.0)	0.313
Body mass index (kg/m^2^)	26.8 ± 3.9	27.0 ± 3.9	0.521
Number of measurements	44.8 ± 24.4	45.4 ± 26.0	0.772
SD of BMI (kg/m^2^)	1.0 ± 0.5	1.2 ± 0.6	<**0.001**
Average SBP (mmHg)	135.3 ± 9.6	141.3 ± 10.7	<**0.001**
Number of measurements	61.8 ± 27.6	68.6 ± 34.1	**0.004**
SD of SBP (mmHg)	13.3 ± 2.6	19.5 ± 3.3	<**0.001**
Maximum of SBP (mmHg)	170.1 ± 15.9	188.4 ± 15.1	<**0.001**
Delta SBP (mmHg)	65.1 ± 20.0	96.0 ± 27.1	<**0.001**
Average DBP (mmHg)	73.1 ± 6.0	74.7 ± 7.1	**0.002**
SD of DBP (mmHg)	6.8 ± 1.5	9.7 ± 2.3	<**0.001**
Maximum of DBP (mmHg)	91.3 ± 9.4	101.9 ± 12.9	<**0.001**
Delta DBP (mmHg)	34.4 ± 11.6	50.0 ± 16.7	<**0.001**
Hemoglobin A_1c_ (%)	7.5 ± 1.0	7.7 ± 1.1	**0.019**
Number of measurements	35.4 ± 10.7	32.0 ± 11.3	<**0.001**
SD of Hemoglobin A_1c_ (%)	0.9 ± 0.6	1.1 ± 0.5	<**0.001**
Total cholesterol (mg/dL)	192.3 ± 26.7	197.2 ± 33.7	**0.034**
Number of measurements	12.0 ± 5.8	11.3 ± 6.9	0.147
High-density lipoprotein (mg/dL)	38.5 ± 10.7	38.4 ± 10.5	0.877
Low-density lipoprotein (mg/dL)	118.6 ± 19.7	118.4 ± 21.0	0.877
Triglyceride (mg/dL)	147.0 ± 106.8	159.8 ± 127.0	0.1640
Average creatinine	0.89 ± 0.4	1.3 ± 1.2	<**0.001**
Number of measurements	13.6 ± 5.1	15.8 ± 9.5	<**0.001**
SD of creatinine	0.1 ± 0.3	0.3 ± 0.4	<**0.001**

Clinical events during the 10-year follow-up period			
PAD (%)	64 (10.2)	50 (25.1)	<**0.001**
Interval from DM diagnosis (y)	8.1 ± 2.0	7.7 ± 2.5	0.403
CVD^a^ (%)	73 (11.7)	50 (25.1)	<**0.001**
Interval from DM diagnosis (y)	5.4 ± 2.8	4.9 ± 3.5	0.388
CAD or MI (%)	24 (3.8)	14 (7.0)	**0.051**
Interval from DM diagnosis (y)	5.4 ± 2.7	3.7 ± 3.3	0.094
TIA or stroke (%)	51 (8.1)	37 (18.6)	<**0.001**
Interval from DM diagnosis (y)	5.3 ± 2.9	5.4 ± 3.4	0.903
Recurrent TIA or stroke events	0.2 ± 0.5	0.4 ± 0.8	0.152
Total follow-up (months)	148.6 ± 15.2	146.4 ± 18.4	0.098

DM, diabetes mellitus; SBP, systolic blood pressure; DBP, diastolic blood pressure; SD, standard deviation; PAD, peripheral arterial disease; CVD, cerebrovascular disease; CAD, coronary artery disease; MI, myocardial infarction; TIA, transient ischemic attack; CKD, chronic kidney disease.

^a^Defined as cerebrovascular disease, neurodegenerative disease, and parkinsonism that required medical treatment and long-term follow-up.

**Table 4:** Multivariate Cox regression analysis of factors associated with the occurrence of peripheral arterial disease.

	Hazard ratio	95% CI	*P* value
Age (+1 y)	1.064	1.043–1.084	<0.001
Nonsmoking	1		
Former smoking	1.645	0.771–2.783	0.244
Current smoking	1.803	1.160–2.804	0.009
High SBP and DBP visit-to-visit variability	1.679	1.141–2.472	0.009
Mean Creatinine (mg/dL)	1.208	1.042–1.401	0.012

DM, diabetes mellitus; SBP, systolic blood pressure; DBP, diastolic blood pressure; CI, confidence interval.
